# CD47 associates with alpha 5 integrin and regulates responses of human articular chondrocytes to mechanical stimulation in an *in vitro *model

**DOI:** 10.1186/ar2350

**Published:** 2008-01-10

**Authors:** Mahmoud Orazizadeh, Herng Sheng Lee, Bianca Groenendijk, S Jane Millward Sadler, Malcolm O Wright, Frederik P Lindberg, Donald M Salter

**Affiliations:** 1 Department of Anatomical Sciences, Medical School, Ahwaz Jondishapour University of Medical Sciences, Ahwaz, Iran; 2 Department of Pathology, Tri-Service General Hospital and National Defense Medical Center, No.325, Sec.2, Chenggong Rd, Neihu District, Taipei City 114, Taiwan; 3 The Division of Pathology, School of Molecular and Clinical Medicine, College of Medicine and Veterinary Medicine, Edinburgh University, 47 Little France Crescent, Edinburgh, EH16 4TJ, UK; 4 Infectious Diseases Division, Department of Medicine, Washington University School of Medicine, 660 S. Euclid Ave, St. Louis, MO, 63110, USA; 5 Centre for Inflammation Research, C2.22 Queen's Medical Research Institute, 47 Little France Crescent, Edinburgh, EH16 4TJ, UK

## Abstract

**Background:**

Recent studies provide evidence of roles for integrins in mechanical signalling in bone and cartilage. Integrin signalling is modulated by various mechanisms, including interaction with other transmembrane proteins. We aimed to identify whether one such protein, integrin-associated protein (CD47/IAP), is expressed by chondrocytes and whether it may regulate integrin-dependent mechanotransduction.

**Methods:**

Chondrocytes, isolated from macroscopically normal and osteoarthritic articular cartilage of human knee joints, were studied in a resting state or following mechanical stimulation at 0.33 Hz. CD47/IAP expression and associations were confirmed by immunohistology, reverse transcription-polymerase chain reaction, Western blotting, and immunoprecipitation. Roles in mechanotransduction were studied by assessing effects of function-blocking antibodies on a range of electrophysiological, cellular, and molecular responses of primary chondrocytes and responses of CD47/IAP-null cell lines transfected with CD47/IAP.

**Results:**

Human articular chondrocytes were shown to express CD47/IAP, predominantly the type 2 isoform. Immunoprecipitation showed association of CD47/IAP with α5 integrin and thrombospondin but not SIRPα (signal-regulatory protein-alpha). The function-blocking anti-CD47/IAP antibody Bric 126 inhibited changes in membrane potential, tyrosine phosphorylation, and elevation of relative levels of aggrecan mRNA induced by mechanical stimulation, whereas in the presence of B6H12, an antibody that has partial agonist activity, a membrane depolarisation rather than a membrane hyperpolarisation response was induced by mechanical stimulation. CD47-null cell lines did not show changes in cell membrane potential following mechanical stimulation. Changes in cell membrane potential following mechanical stimulation were seen when CD47-null cells were transfected with CD47/IAP expression vectors but were not seen following mechanical stimulation of cells transfected with vectors for the extracellular immunoglobulin variable (IgV) domain of CD47/IAP in the absence of the transmembrane and intracellular domains.

**Conclusion:**

CD47/IAP is necessary for chondrocyte mechanotransduction. Through interactions with α5β1 integrin and thrombospondin, CD47/IAP may modulate chondrocyte responses to mechanical signals.

## Introduction

Structural integrity of articular cartilage is dependent on physical loading and joint movement. Overloading and unloading are associated with proteoglycan depletion leading to osteoarthritis (OA), whereas proteoglycan synthesis and articular cartilage thickness are increased by mechanical stresses associated with physiological levels of exercise [1-3]. The mechanisms by which mechanical forces regulate chondrocyte function are beginning to be defined and appear to involve the activation of a variety of intracellular signalling pathways, at least some of which require integrin-mediated events. In normal human articular chondrocytes, cyclical mechanical stimulation at 0.33 Hz (2 seconds on, 1 second off) *in vitro *results in activation of a mechanotransduction pathway that ultimately leads to upregulation of aggrecan gene expression and downregulation of matrix metalloproteinase-3 gene expression [4]. The mechanotransduction pathway appears to be dependent on α5β1 integrin signalling, stretch-activated ion channels, the actin cytoskeleton, and subsequent secretion of interleukin-4, which (via a paracrine/autocrine signalling loop) results in the activation of the enzymes phospholipase C and protein kinase C, and the production of inositol triphosphate [5-7]. In contrast, although mechanical stimulation activates α5β1-mediated signalling events, chondrocytes from osteoarthritic cartilage do not show alteration in levels of aggrecan or matrix metalloproteinase-3 mRNA following 0.33-Hz mechanical stimulation [4,8]. The reasons why normal and OA chondrocyte mechanotransduction through α5β1 integrin differ are unclear. However, previous experiments undertaken with human bone cells, in which antibodies to the integrin-associated protein (CD47/IAP) had different effects on integrin-dependent responses, suggest critical roles for this molecule in mechanotransduction [9].

CD47/IAP is a 45- to 55-kDa plasma membrane protein that is physically and functionally associated with integrins [10-13]. CD47/IAP has a heavily glycosylated extracellular immunoglobulin variable (IgV)-like domain, a domain containing multiple membrane-spanning segments, and a short cytoplasmic tail. Four alternatively spliced forms that differ in the length of the cytoplasmic tail have been identified [14]. CD47/IAP has a broad tissue expression and has been identified as a receptor for thrombospondin (TSP) family members [15] and signal-regulatory protein-alpha (SIRPα) [16]. The roles of CD47/IAP are being elucidated and it is becoming increasingly clear that it has important roles in regulation and modulation of integrin signalling [14,17-21]. CD47/IAP is found in association with αVβ3 and other integrins [10-13]. Antibodies to CD47/IAP have been shown to block the increase in intracellular calcium which occurs upon endothelial cell adhesion to fibronectin-coated surfaces without affecting cell adhesion to those surfaces and to inhibit activation of neutrophil polymorphonucleocyte activity, including phagocytosis, respiratory burst, and transendothelial and transepithelial chemotaxis induced by arginine-glycine-aspartic acid (RGD) containing synthetic peptides and protein [17]. Ligation of CD47/IAP on melanoma cells results in modulation of αVβ3 function [18]. CD47/IAP associates with α2β1 integrin on vascular smooth muscle cells and can modify the function of this integrin [19]. Furthermore, ligation of CD47/IAP to αVβ3 ligation inhibits α5β1 and αVβ5 integrin-dependent phagocytosis (F.P. Lindberg, unpublished observations). The molecular mechanisms by which CD47/IAP regulates integrin-mediated events are being elucidated, and studies suggest that CD47/IAP ligation may lead to modulation of integrin conformation and affinity state or influence integrin-mediated signal transduction at a further downstream level via a Gi-type heterotrimeric G protein [20-22]. This study was undertaken to establish whether CD47/IAP is expressed by human articular chondrocytes and to establish whether it and potential ligands such as TSP and SIRPα have roles in integrin-dependent responses of human articular chondrocytes to mechanical stimulation.

## Materials and methods

### Source of tissue, chondrocyte culture, and cell lines

Human articular cartilage was obtained with ethical approval from the Lothian Research Ethics Committee and patients' consent at operation from knee joint arthroplasty specimens and amputations for peripheral vascular disease. Cartilage was assessed macroscopically for the presence or absence of osteoarthritic changes and graded for OA using the Collins/McElligott system. Chondrocytes were isolated by sequential enzyme digestion, and cells were seeded in Iscove's modified Dulbecco's medium (Gibco, now part of Invitrogen Corporation, Carlsbad, CA, USA) supplemented with 10% fetal calf serum (Sigma-Aldrich, St. Louis, MO, USA), 100 IU/mL penicillin (Invitrogen Corporation), and 100 μg/mL streptomycin (Invitrogen Corporation) to a final density of 5 × 10^5^/mL. Primary, non-confluent, 1- to 2-week cultures of chondrocytes were used in all experiments. The day before mechanical stimulation, culture media containing serum was replaced by serum-free media. In some experiments, cell lines were used. OV10 cells, an ovarian carcinoma cell line lacking CD47/IAP, were stably transfected with human CD47/IAP form 2 cDNA in the absence (315 – CD47-2) or presence (315/164 – β_3_/CD47-2) of β_3 _cDNA or with CD47's extracellular IgV domain linked to a glycosylphosphatidylinositol anchor (OV10 148A10T – CD47IgV-GPI) [22]. 3657L cells are polyclonal CD47^+ ^fibroblasts derived from B6 mice. 3656L cells are lung fibroblasts derived from CD47/IAP-null mice [23] and transfected with mouse (1/171) or human (1/315) CD47/IAP form 2 cDNA or vector control (1/Sra).

### Antibodies

The following primary antibodies were used in electrophysiology experiments, immunohistology, and immunoprecipitation/Western blotting as indicated. Anti-CD47/IAP – Bric 126 (International Blood Group Reference Laboratory, Bristol, UK), CC2C6 (Hans-Jörg Büring, University of Tübingen, Germany), B6H12, miap 400.1, and 2B7 (Washington University School of Medicine, St. Louis, MO, USA), goat polyclonal anti-CD47 (Santa Cruz Biotechnology, Inc., Santa Cruz, CA, USA), anti-CD49e/α5 integrin – Sam-1 (Serotec Ltd., Oxford, UK), anti-CD29/β1 integrin clone JB1A (Chemicon International, Temecula, CA, USA), anti-TSP clone P10 (Chemicon International), anti-SIRPα (rabbit polyclonal antibody; ABR, Affinity BioReagents, Inc., Golden, CO, USA), anti-HRP (phosphotyrosine-horseradish peroxidase) conjugated (Amersham Life Sciences, now part of GE Healthcare, Little Chalfont, Buckinghamshire, UK), and anti-focal adhesion kinase (Santa Cruz Biotechnology, Inc.).

### Immunohistochemistry

Samples of normal articular cartilage (Collins grade 0) obtained from 1 female (age 67 years) and 7 males (median age 71 years, range 53 to 88 years) and osteoarthritic cartilage (Collins grade 1 to 3) obtained from 13 males (median age 71 years, range 53 to 88 years) were snap-frozen in liquid nitrogen. Sections (4 μm) were cut with a Bright cryostat (Bright Instrument Co. Ltd., Huntingdon, UK), mounted on poly-L-lysine-coated glass slides, allowed to come to room temperature, and fixed with acetone for 10 minutes. Sections were stained with Bric 126 by an avidin-biotin-immunoperoxidase technique. For negative control, the primary antibody was replaced by non-immune mouse immunoglobulin.

### Protein extraction, Western blotting, and immunoprecipitation

The methods for protein extraction, immunoprecipitation, and Western blotting used have been described previously [7]. Cells at rest or following mechanical stimulation were washed with ice-cold phosphate-buffered saline containing 100 μM Na_3_VO_4 _(Sigma-Aldrich) and lysed *in situ *with ice-cold lysis buffer containing 1% Igepal (Sigma-Aldrich), 100 μM Na_3_VO_4_, and protease inhibitor cocktail tablet (Boehringer Ingelheim GmbH, Ingelheim, Germany) at 4°C for 15 minutes. Supernatants were collected after centrifugation at 13,000 rpm for 15 minutes. For CD47/IAP immunoprecipitation, a 1-mL aliquot of protein at a concentration of 500 μg/mL was incubated at 4°C with either Bric 126 or CC2C6 for 1 hour and then with protein A-Sepharose (Pharmacia-LKB Biotechnology, Uppsala, Sweden) for 1 hour. Whole-cell extracts or immunoprecipitated proteins were separated on a 7.5% SDS-PAGE under reducing conditions. Following electrophoresis, immunoprecipitated proteins or whole-cell lysates were transferred onto polyvinylidene fluoride membranes (Immobilon-P; Millipore Corporation, Billerica, MA, USA, and Sigma-Aldrich). Membranes were blocked overnight at 4°C with 2% bovine serum albumin (BSA) in TBST (12.5 mM Tris/HCl, pH 7.6, 137 mM NaCl, 0.1% Tween 20). After washing with TBST, blots were incubated for 1 hour at room temperature with primary antibodies and then HRP-labelled secondary antibodies. Membranes were rewashed extensively and bands were detected using Enhanced Chemiluminescense Plus (GE Healthcare). For immunoprecipitation of phosphotyrosine, monoclonal antiphosphotyrosine agarose beads (Sigma-Aldrich) were used.

### Electrophysiological measurements and mechanical stimulation

Membrane potentials of cells were recorded using a single electrode bridge circuit and calibrator [4,8,9]. Membrane potentials of cells were measured and results were accepted if, on cell impalement, any rapid changes in voltage to the membrane potential level remained constant for at least 30 seconds. The membrane potentials of 5 to 10 cells were measured prior to and following addition of the reagent to be tested and/or mechanical stimulation. Each experiment was undertaken at least three times on cells from different donors. The technique and apparatus used for mechanical stimulation of primary human articular chondrocytes have been described in detail [4]. Plastic tissue culture dishes (diameter 55 mm) (NUNC A/S, Roskilde, Denmark) containing sparse primary monolayer cultures of human articular chondrocyte monolayer were placed in a sealed pressure chamber with inlet and outlet ports. The chamber was pressurised using helium gas from a cylinder at a frequency determined by an electronic timer that controlled the inlet and outlet valves. The standard stimulation regime used was a frequency of 0.33 Hz (2 seconds on, 1 second off) for 20 minutes at 37°C at a pressure of 16 kPa above atmospheric pressure. This system produces 3700 microstrain on the base of the culture dish.

### Reverse transcription-polymerase chain reaction and gel analysis

Total RNA was extracted from cultured chondrocytes using a denaturing buffer of 4 M guanidine thiocyanate, 0.75 M sodium citrate, 10% (wt/vol) lauroyl sarcosine, and 7.2 μL/mL β-mercaptoethanol. Prior to cDNA synthesis, all RNA samples were incubated with DNAse I (Life Technologies, now part of Invitrogen Corporation) for 15 minutes in the presence of an RNAse inhibitor (Pharmacia, now part of GE Healthcare). Template cDNA was synthesised using 0.5 μg of RNA, Superscript II, and oligo dT (12–18) (Invitrogen Corporation) according to the manufacturer's instructions. The primers used for the polymerase chain reactions (PCRs) were (upstream/downstream) CD47/IAP, 5'-GTTGGACTGAGTCTCTGTATTGCGGCGTGT-3' and 5'-CACAAGTGTATTCCTTTCACGTCTTACTACTC-3'; glyceraldehyde-3-phosphate dehydrogenase (GAPDH), 5'-CCACCCATGGCAAATTCCATGGCA-3' and 5'-TCTAGACGGCAGGTCAGGTCCACC-3'; and aggrecan, 5'-TGAGGAGGGCTGGAACAAGTACC-3' and 5'-GGAGGTGGTAATTGCAGGGAACA-3'.

A typical 20-μL PCR contained 20 mM ammonium sulphate, 75 mM Tris-HCl, pH 8.8, 0.01% (vol/vol) Tween-20, 1 μM each primer, 2 μL of cDNA, 100 μM dNTPs, 0.1% (wt/vol) BSA, and 0.25 U Taq polymerase (BioGene Ltd., Kimbolton, UK). The magnesium chloride concentrations for each primer pair were 2.5 mM (GAPDH) and 1.25 mM (aggrecan), and the following programme was used for aggrecan reactions: 94°C for 3 minutes, 24 cycles of 94°C for 1 minute, 60°C for 1 minute, and 72°C for 1 minute 30 seconds. PCR products were analysed by electrophoresis using a 1% (wt/vol) agarose gel stained with ethidium bromide, and the intensity of each band was measured under UV fluorescence using EASY Image analysis software (Scotlab Ltd., Coatbridge, UK). The ratio of intensities of the bands for the aggrecan product compared with the housekeeping gene *GAPDH *was calculated and compared. Each donor was tested in duplicate, and at least three donors were used for each experiment.

### Statistics

The mean, standard deviation, and standard error of the mean were calculated for each time point. The Student *t *test was used to evaluate whether there was statistical significance between each of the points calculated.

## Results

### Expression of CD47/IAP in human articular cartilage and extracted chondrocytes

**Figure 1:**
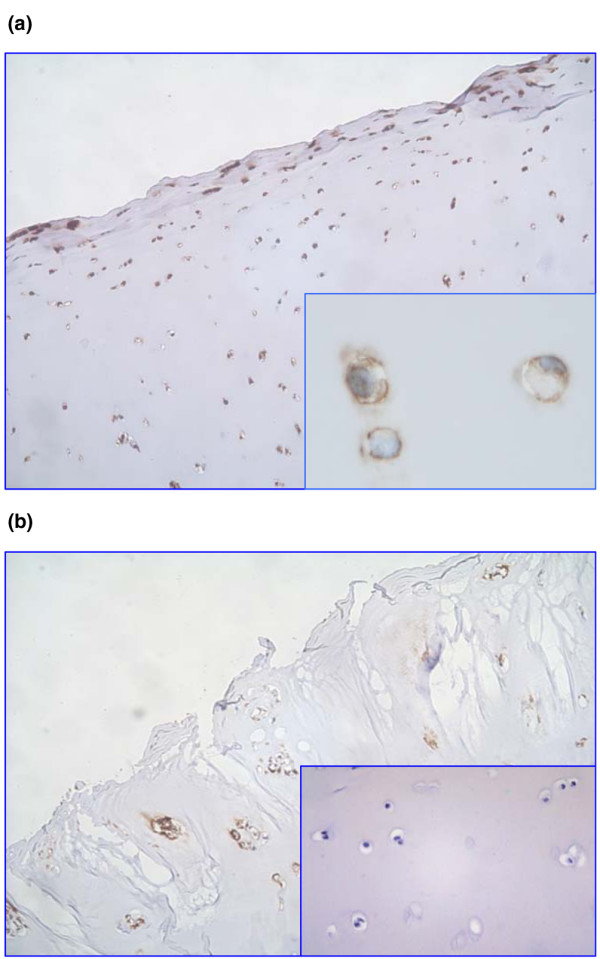
Expression of CD47/IAP in human articular cartilage. Sections of human articular cartilage were stained with the anti-CD47 antibody Bric 126, resulting in strong labelling of resident chondrocytes. **(a) **Normal articular cartilage (original magnification ×50). Inset: high-power view (original magnification ×400). **(b) **Osteoarthritic articular cartilage (original magnification ×50). Inset: negative control non-immune mouse immunoglobulin (original magnification ×100). IAP, integrin-associated protein.

**Figure 2:**
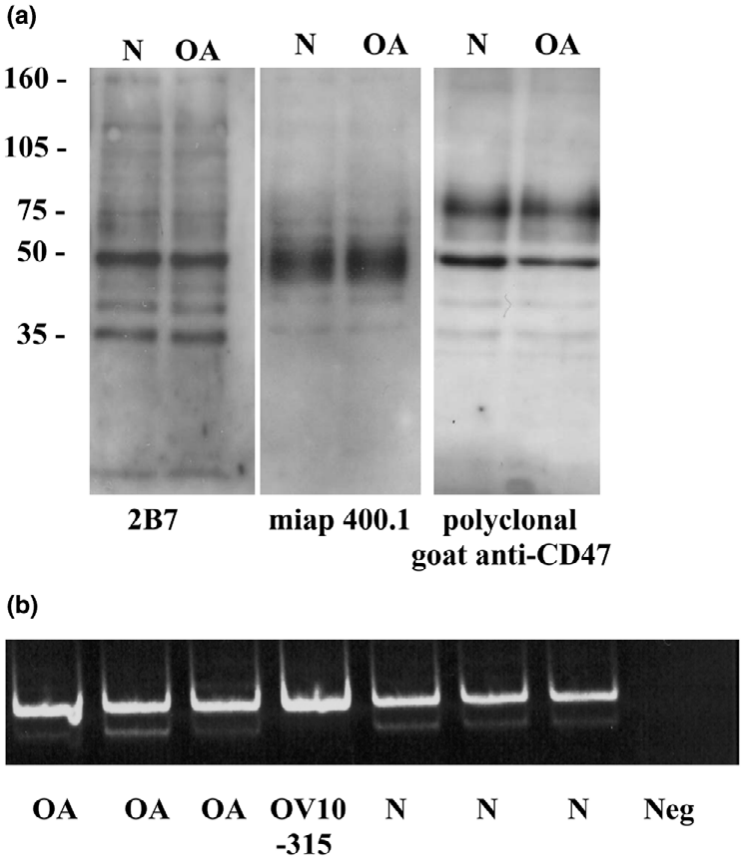
Expression of CD47/IAP by human articular chondrocytes. **(a) **Western blotting of protein extracts from human articular chondrocytes with three different anti-CD47/IAP antibodies (monoclonal antibodies 2B7, miap 400.1, and a goat polyclonal anti-CD47/IAP) showing a predominant band of approximately 50 kDa in protein extracts from chondrocytes extracted from both normal and osteoarthritic chondrocytes. **(b) **Reverse transcription-polymerase chain reaction on RNA extracted from primary cultures of chondrocytes from normal and osteoarthritic chondrocytes undertaken with specific primers for CD47/IAP showing predominant expression of the type 2 isoform. IAP, integrin-associated protein; OA, osteoarthritic human articular chondrocytes; OV10-315, CD47 negative ovarian carcinoma cell line transfected with human CD47 type 2 isoform; N, normal human articular chondrocytes; Neg, negative control (reverse transcription step omitted).

The vast majority of chondrocytes in both normal (*n *= 8) and osteoarthritic (*n *= 13) cartilage were strongly stained with antibodies to CD47/IAP (Figure 1a,b). There was no apparent difference in immunoreactivity between chondrocytes in normal and osteoarthritic cartilage. Similarly, no difference in expression of CD47/IAP was seen when protein extracts from primary monolayer cultures of chondrocytes derived from normal and osteoarthritic cartilage were immunoblotted with a panel of three different anti-CD47/IAP antibodies. In both normal and OA chondrocytes, CD47/IAP was identified as a molecule of approximately 50 kDa molecular weight (Figure 2a). Reverse transcription-polymerase chain reaction from extracted RNA resulted in the production of two products, with a predominant product consistent with expression of the type 2 isoform of CD47/IAP (Figure 2b). The cell line OV10-315 is transfected with CD47/IAP type 2 DNA and therefore expresses only this isoform.

### Molecular associations of CD47/IAP when expressed by human articular chondrocytes

**Figure 3:**
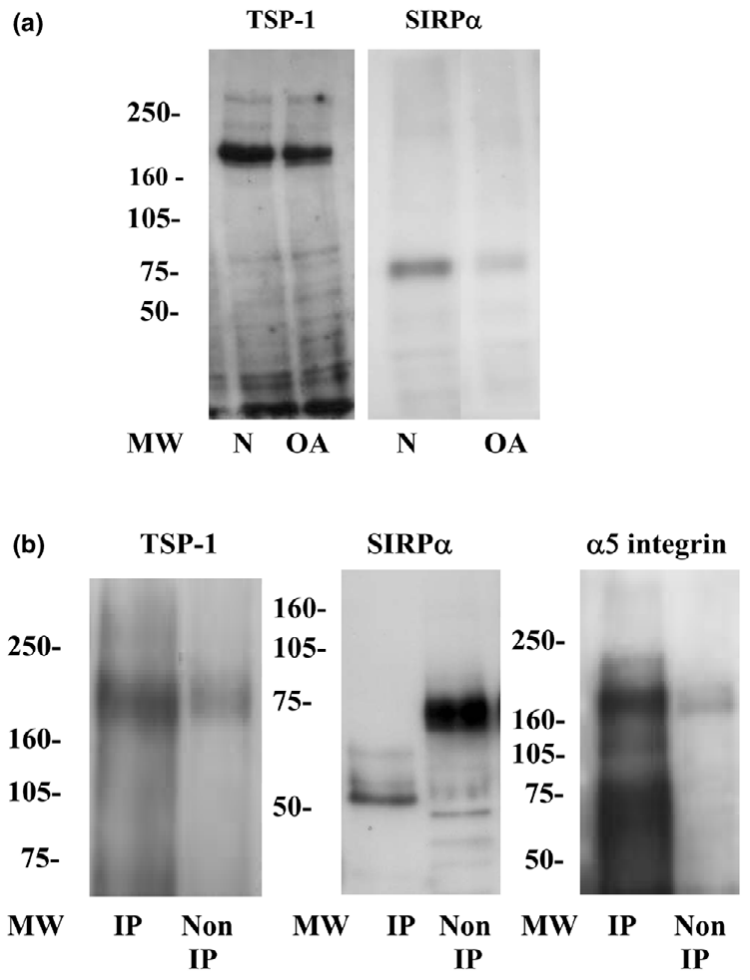
Expression of thrombospondin-1 (TSP-1) and signal-regulatory protein-alpha (SIRPα) by human articular chondrocytes and molecular associations of CD47/IAP. **(a) **Protein extracts from primary cultures of human articular chondrocytes were separated by SDS-PAGE and immunoblotted with antibodies against TSP-1 and SIRPα. For TSP-1, a 6% gel under reducing conditions was used, whereas for SIRPα, an 8% gel under non-reducing conditions was used. **(b) **Protein extracts from primary cultures of human articular chondrocytes were immunoprecipitated with antibodies against CD47/IAP and immunoblotted for TSP-1, SIRPα, or α5 integrin. TSP-1, SIRPα, and α5 integrin are identified in non-immunoprecipitated extracts, but only TSP-1 and α5 integrin co-immunoprecipitate with CD47/IAP. IP, immunoprecipitated protein extracts; MW, molecular weight; N, normal human articular chondrocytes; Non IP, non-immunoprecipitated protein extracts; OA, osteoarthritic human articular chondrocytes.

CD47/IAP has been shown to be associated with a number of different extracellular and membrane proteins, including integrins, TSPs, and SIRPα, in a variety of cell types. To establish whether similar associations were occurring in human articular chondrocytes, a series of experiments were undertaken to show the presence of potential ligands and to investigate possible interactions. Protein extracts from primary cultures of human articular chondrocytes were first immunoblotted for TSP-1 and SIRPα, and expression of these molecules by human articular chondrocytes was confirmed (Figure 3a). Protein extracts from human articular chondrocytes were then immunoprecipitated with anti-CD47/IAP, and the precipitated proteins were subjected to gel electrophoresis and Western blotting with antibodies specific for TSP-1, SIRPα, and α5 integrin. TSP-1 and α5 integrin, but not SIRPα, were shown to immunoprecipitate with CD47/IAP (Figure 3b). Immunoprecipitation of α5 integrin and β1 integrin and subsequent Western blotting showed co-precipitation of CD47/IAP with α5 integrin precipitates only (results not shown).

### Roles for CD47/IAP in regulation of chondrocyte mechanotransduction

To investigate potential roles for CD47/IAP in chondrocyte mechanotransduction, chondrocytes were subjected to cyclical mechanical stimulation and the responses of the cells assessed by measuring either changes in membrane potential, changes in protein tyrosine phosphorylation, or changes in the relative levels of aggrecan mRNA within cells.

### Effects on cell membrane potential

Twenty minutes of mechanical stimulation of normal human articular chondrocytes at 0.33 Hz results in a significant and reproducible membrane hyperpolarisation. In contrast, chondrocytes from osteoarthritic cartilage show a membrane depolarisation response. Previous studies have shown that these responses are the result of activation of a mechanotransduction pathway that is α5β1-integrin-dependent [3,8]. In normal chondrocytes, mechanical signal results in activation of SK (small conductance) calcium-activated potassium channels, with potassium efflux and cell membrane hyperpolarisation. In osteoarthritic chondrocytes, the mechanotransduction pathway leads to activation of tetrodotoxin-sensitive channels, sodium influx, and cell membrane depolarisation. To establish whether CD47/IAP has roles in the response of articular chondrocytes to 0.33-Hz mechanical stimulation, chondrocytes were mechanically stimulated in the presence of the function-modifying anti-CD47/IAP antibodies Bric 126 and B6H12 (Table 1). The addition of Bric 126 and B6H12 to articular chondrocytes 10 minutes prior to the period of mechanical stimulation had no significant effect on the resting cell membrane potential. In the presence of Bric 126, both the membrane hyperpolarisation response of normal chondrocytes and the membrane depolarisation response of osteoarthritic chondrocytes to 0.33-Hz mechanical stimulation were blocked. When normal chondrocytes were mechanically stimulated in the presence of B6H12, an anti-CD47/IAP antibody that has partial agonist activity [24,25], a membrane depolarisation response was seen.

**Table 1:** Effect of anti-CD47/IAP, anti-TSP-1, and anti-SIRPα on mechanically induced changes in human articular chondrocyte membrane potential

		Membrane potential (-mV) (mean ± SEM)			
Sample	Reagent	Resting	Reagent alone	Reagent + 0.33 Hz	Percentage change

Normal chondrocytes	Nil	25.0 ± 0.4	**-**	30.2 ± 0.6	+21^a^
	Bric 126	26.2 ± 0.8	27.2 ± 0.4	27.6 ± 1.2	+2^b^
	B6H12	25.8 ± 2.2	26.3 ± 1.9	17.1 ± 1.0	-35^a^
Osteoarthritic chondrocytes	Nil	30.0 ± 1.9	**-**	17.4 ± 1.3	-42^a^
	Bric 126	28.0 ± 1.6	28.0 ± 2.1	27.4 ± 1.8	-2^b^
	Anti-TSP-1	29.6 ± 1.2	29.0 ± 0.8	29.8 ± 1.6	+3^b^
	SE5A5	29.2 ± 1.0	29.0 ± 1.1	23.2 ± 0.8	-20^a^

Membrane potentials of chondrocytes were measured at rest in the absence or presence of the appropriate antibody and after 20 minutes of mechanical stimulation at 0.33 Hz. Bric 126 is a function-blocking anti-CD47/IAP antibody. B6H12 is an anti-CD47/IAP antibody that has partial agonist activity. SE5A5 is an anti-SIRPα antibody. *N *= 5. ^a^*p *< 0.001; ^b^not significant. IAP, integrin-associated protein; SEM, standard error of the mean; SIRPα, signal-regulatory protein-alpha; TSP-1, thrombospondin-1.

Similar experiments were undertaken with chondrocytes from osteoarthritic cartilage and antibodies to TSP-1 and SIRPα (Table 1). Neither the anti-TSP-1 antibody nor the anti-SIRPα antibody SEAA5 had significant effects on the resting membrane potential of chondrocytes. When OA chondrocytes were mechanically stimulated at 0.33 Hz in the presence of the anti-TSP-1 antibody, no change in cell membrane potential was seen, indicating blockade of the mechanotransduction pathway that results in membrane depolarisation. In contrast, in the presence of SE5A5, an antibody against SIRPα, a statistically significant change in membrane potential – membrane depolarisation – was seen following mechanical stimulation. The degree to which the membrane potential changed following mechanical stimulation in the presence of SE5A5, however, was less than that seen in the absence of antibody, raising the possibility of a partial blocking effect.

### Effects on protein tyrosine phosphorylation

**Figure 4:**
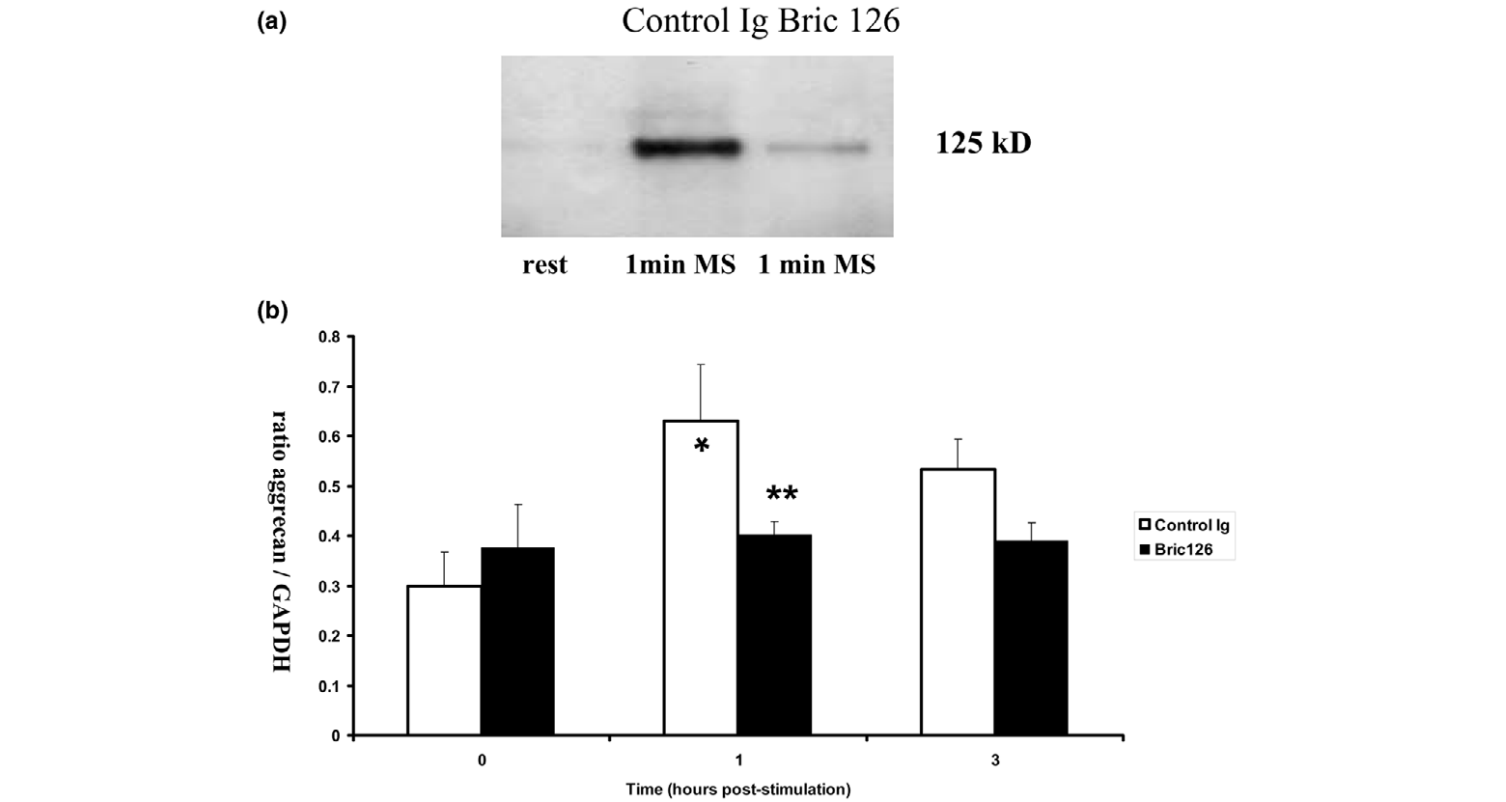
Effect of anti-CD47/IAP antibody on chondrocyte responses to mechanical stimulation. **(a) **Cell lysates from normal human articular chondrocytes at rest or mechanically stimulated at 0.33 Hz for 1 minute in the presence of either non-immune mouse immunoglobulin (Ig) or the function-blocking CD47/IAP antibody Bric 126 were immunoprecipitated with anti-phosphotyrosine and immunoblotted with mouse monoclonal antibody anti-phosphotyrosine-horseradish peroxidase, PY-20, at 1:1,000. Identical amounts of whole-cell lysates were used for immunoprecipitation of phosphotyrosinated proteins from stimulated and unstimulated cells. Tyrosine phosphorylation of a 125-kDa protein is increased after 1 minute of mechanical stimulation but this response is diminished by the presence of the anti-CD47/IAP antibody Bric 126. **(b) **Following 0.33-Hz mechanical stimulation for 20 minutes in the presence of control non-immune mouse Ig or anti-CD47/IAP antibody Bric 126, relative levels of aggrecan mRNA were assessed in cells immediately and 1 and 3 hours after incubation at 37°C. Consistent with previous results [7], mechanical stimulation of normal articular chondrocytes in the presence of non-immune mouse Ig results in an increase in relative levels of aggrecan mRNA. This response is inhibited by the presence of the anti-CD47/IAP antibody Bric 126. *p < 0.05, 1 hour after mechanical stimulation versus immediate post stimulation. **p < 0.05, 1 hour after stimulation in the presence of Bric 126 versus 1 hour after stimulation in the presence of non-immune mouse Ig. GAPDH, glyceraldehyde-3-phosphate dehydrogenase; IAP, integrin-associated protein; MS, mechanical stimulation.

Mechanical stimulation of normal human articular chondrocytes at 0.33 Hz results in increased tyrosine phosphorylation of a protein of approximately 125 kDa within 1 minute. This response is inhibited by the presence of anti-CD47/IAP monoclonal antibody Bric 126 (Figure 4a). Bric 126 has no direct effect on tyrosine phosphorylation in the absence of mechanical stimulation (results not shown).

### Effects on relative levels of aggrecan mRNA

Following mechanical stimulation at 0.33 Hz for 20 minutes, relative levels of aggrecan mRNA are elevated in normal articular chondrocytes at 1 and 3 hours after stimulation (Figure 4b; *p *< 0.05). In the presence of the anti-CD47/IAP monoclonal antibody Bric 126, no changes in the aggrecan mRNA ratio relative to the housekeeping gene *GAPDH *are seen following mechanical stimulation (Figure 4b; *p *> 0.05). Bric 126, in the absence of mechanical stimulation, had no effect on relative levels of aggrecan mRNA.

### CD47/IAP is necessary for mechanical signalling

To ascertain whether CD47/IAP is required for mechanical signalling at different frequencies of stimulation, CD47/IAP-null cells (OV10, human carcinoma cell lines, and mouse CD47/IAP^-/- ^lung fibroblasts) and cells transfected with CD47/IAP type 2 DNA or vector controls were used and the electrophysiological membrane response was assessed. The results are shown in Table 2. 3657L cells, lung fibroblasts derived from wild-type CD47/IAP-expressing mice, showed a membrane depolarisation response when mechanically stimulated at 0.33 Hz. The same cells, however, showed a membrane hyperpolarisation response when mechanically stimulated at 0.083 Hz. 3656 1/Sra cells, CD47/IAP^-/- ^mouse lung fibroblasts derived from CD47/IAP knockout mice transfected with vector control, showed no change in cell membrane potential when mechanically stimulated at both 0.33 and 0.083 Hz. CD47/IAP^-/- ^mouse lung fibroblasts transfected with either mouse (3656 1/171) or human (3656 1/315) CD47/IAP type 2 DNA showed a membrane hyperpolarisation response following stimulation at 0.083 Hz and a membrane depolarisation response following mechanical stimulation at 0.33 Hz.

**Table 2:** Effect of CD47/IAP transfection on the electrophysiological response of CD47-null cells to mechanical stimulation

		Membrane potential (-mV) (mean ± SEM)		
Frequency of stimulation	Cell (phenotype)	Resting	Post mechanical stimulation	Percentage change

0.33 Hz	3657L (CD47 wild-type+)	10.9 ± 0.27	4.5 ± 0.64	-59^a^
	3656 1/Sra (CD47-null)	14.0 ± 1.36	13.8 ± 0.46	-1^b^
	3656 1/171 (mouse CD47 type 2+)	13.1 ± 0.52	5.9 ± 0.65	-55^a^
	3656 1/315 (human CD47 type 2+)	11.8 ± 1.03	4.6 ± 1.03	-61^a^
				
0.083 Hz	3657L (CD47 wild-type+)	10.8 ± 0.6	11.9 ± 0.72	+10^b^
				
	3656 1/Sra (CD47-null)	12.4 ± 0.43	13.0 ± 0.27	+5^b^
	3656 1/171 (mouse CD47 type 2+)	12.8 ± 0.53	20.4 ± 0.92	+48^a^
	3656 1/315 (human CD47 type 2+)	12.2 ± 0.91	17.2 ± 0.32	+42^a^
				
	OV10 58A2 (CD47-null)	10.2 ± 0.85	10.8 ± 1.50	+6^b^
	OV10 315 (human CD47 type 2+)	7.0 ± 0.84	18.6 ± 1.86	+165^a^
	OV10 164/315 (β3/CD47 type 2+)	35.4 ± 1.6	49.2 ± 3.34	+39^a^
	OV10 148A10T (CD47IgV/GPI)	22.6 ± 1.33	22.2 ± 1.07	0^b^

CD47-null cells or cells transfected with either type 2 CD47 cDNA, CD47 extracellular immunoglobulin variable (IgV) domain linked to a glycosylphosphatidylinositol anchor, or vector control were studied. Membrane potentials of cells were measured at rest and after 20 minutes of mechanical stimulation at 0.33 or 0.083 Hz. *N *= 5. ^a^*p *≤ 0.01; ^b^not significant; SEM, standard error of the mean.

OV10 cells, derived from a CD47/IAP-negative human carcinoma, showed no significant change in membrane potential when stimulated at 0.083 Hz. Mechanical stimulation (0.083 Hz) of OV10 cells that had been transfected with human CD47/IAP alone or transfected with CD47/IAP in association with β3 integrin resulted in a significant membrane hyperpolarisation of these cells. In contrast, OV10 cells transfected with CD47/IAP extracellular IgV domain linked to a glycosylphosphatidylinositol anchor showed no significant change in cell membrane potential following 0.083-Hz stimulation. These cells, although expressing the extracellular IgV domain of CD47/IAP, do not express the transmembrane domain or the intracytoplasmic tail of the CD47/IAP, indicating that the extracellular domain by itself is insufficient for cellular recognition and response to the mechanical stimulus.

## Discussion

In this study, we have demonstrated expression of CD47/IAP by human articular chondrocytes with predominant expression of the type 2 isoform. The type 2 isoform of CD47/IAP, which has the second shortest cytoplasmic tail, is the most ubiquitously expressed form of this molecule [14]. Co-immunoprecipitation studies showed associations between CD47/IAP and both α5 integrin and TSP-1. CD47/IAP did not co-immunoprecipitate with SIRPα. These results are not surprising as interactions of CD47/IAP and integrins are through a cis mechanism whereby adjacent molecules in the cell membrane of an individual cell may interact. In contrast, CD47/IAP-SIRPα interactions are typically trans in nature, interactions being between molecules on adjacent cells. As chondrocytes do not make cell-cell contact in cartilage, such trans interactions would not be expected. Roles for SIRPα in chondrocytes remain unclear.

Functional roles for CD47/IAP in chondrocyte mechanotransduction are supported by the observations that antibodies to CD47/IAP inhibited the electrophysiological, biochemical, and molecular responses reproducibly induced when human articular chondrocytes are mechanically stimulated at 0.33 Hz in an *in vitro *monolayer culture model system. CD47/IAP is also shown to be necessary for the electrophysiological response of mouse lung fibroblasts and OV10 carcinoma cells to mechanical stimulation.

We have previously demonstrated that α5β1 integrin, the classical fibronectin receptor, has a pivotal role in human articular chondrocyte mechanotransduction, most likely by acting as a mechanoreceptor [4,6,8]. The present study suggests that CD47/IAP also has important roles in regulation of chondrocyte mechanotransduction, potentially through association with α5β1 integrin. CD47/IAP has been shown to co-immunoprecipitate with a number of integrins, including αVβ3, the platelet fibrinogen receptor αIIβ3, the collagen receptor α2β1, and α4β1 in other cell types [18,19,26]. The molecular interactions between CD47/IAP and integrins have best been studied in respect to αVβ3 integrin and suggest that associations between CD47/IAP and αVβ3 integrin are dependent on the extracellular IgV domain of CD47/IAP, the multiple membrane-spanning segment aiding stabilisation of the molecular complex [14,26]. Associations between α5β1 integrin and CD47/IAP are likely to occur through similar mechanisms, and an intact supramolecular complex may be necessary for integrin-mediated mechanotransduction. The observations that an anti-CD47/IAP antibody B6H12 that has partial agonist activity modulated the response to mechanical stimulation would support this idea. This antibody, which is inhibitory for αVβ3 [26] and αIIβ3 [18] integrin functions, activates α5β1- and α4β1-dependent adhesion of CD47/IAP-expressing cells by a process that involves activation of a CD47/IAP α4β1 complex [24,25].

The mechanism by which CD47/IAP influences chondrocyte mechanotransduction is unclear and may involve integrin-dependent [27] and -independent [28] pathways. Previous work in a variety of cell systems supports involvement of Gαi-containing heterotrimeric GTPases in CD47/IAP regulation of integrin activity [29,30]. More recently, a different mechanism by which CD47/IAP can modulate integrin affinity without intracellular signalling has been suggested [31]. The extracellular IgV domain of CD47/IAP may interact with the integrin, inducing change to a high-affinity state presumably by modulating a structural change in the integrin extracellular domain. In the present work, transfection studies were undertaken with a human ovarian carcinoma cell line and a mouse lung fibroblast cell line, rather than human chondrocytes, in an attempt to provide additional understanding of CD47/IAP involvement in cellular mechanotransduction. Although these cells may express integrin profiles different from that of articular chondrocytes and mechanotransduction pathways may be different, the results allow some speculation of how CD47/IAP may function in mechanical signalling. Our observations that OV10 cells expressing the extracellular IgV domain of CD47/IAP attached to the plasma membrane with a glycosylphosphatidylinositol anchor did not show electrophysiological responses to mechanical stimulation suggest that the multiple membrane-spanning domain or the cytoplasmic tail or both are necessary for mechanotransduction. The multiple membrane-spanning domain may act to stabilise associations of CD47/IAP with integrins, whereas the cytoplasmic tail may be involved in regulation of the cytoskeleton through PLICs (proteins linking IAP to cytoskeleton) [32,33]. Alternatively, interactions between CD47/IAP, integrins, and P2Y_2_Rs [34], which recently have been demonstrated to be involved in chondrocyte and bone cell mechanotransduction [35,36], may be important for coupling the P2Y_2_R to G_o_.

Inhibition of the electrophysiological response of chondrocytes by anti-TSP-1 antibodies in addition to the function-blocking anti-CD47/IAP antibody Bric 126 suggests that TSP-CD47/IAP interactions may also be important in chondrocyte responses to mechanical stimulation. CD47/IAP is known to be a receptor for TSPs [15], recognising and binding to the RFYVVMWK sequence expressed in the C-terminal cell-binding domain. TSP-1 and TSP5/cartilage oligomeric matrix protein (COMP) are known to be present in human cartilage [37-39]. TSP5/COMP has roles in collagen fibril formation [40], but functions for cellular-associated TSP in cartilage are not clear. In chondrocytes, a TSP-CD47/IAP complex may associate with α5β1 integrin to modulate alterations in integrin affinity/avidity state critical in the mechanotransduction process. Indeed, the proximity of the RGD and RFYVVMWK motifs in the TSP molecule might allow for simultaneous engagement of α5β1 integrin and CD47/IAP [41]. It has been speculated that the five membrane-spanning segments of CD47/IAP and the two membrane-spanning domains of the heterodimeric integrin form an *ad hoc *seven-transmembrane receptor that, with an adhesive ligand such as TSP, could activate GTPase activity in a manner analogous to that of conventional heptaspanins [41]. Whether such a signalling complex is the basic mechanoreceptor in chondrocytes and is the route through which mechanical stimuli regulate activation of downstream signalling events such as Ras, Rac, and Cdc42 activation [42] and hence modulate gene expression requires further study.

## Conclusion

Articular cartilage is subjected to a wide range of mechanical forces *in vivo *as part of normal joint movement and loading. Mechanical loading within a physiological range is necessary for the maintenance of articular cartilage in a healthy state, whereas overloading or underloading typically results in cartilage degeneration and development of OA. Whether chondrocytes show remodelling, anabolic, or catabolic responses to mechanical stimuli depends on a number of factors, including the nature of the mechanical stimulus, the source of the cartilage, and donor age. *In vitro *studies have demonstrated that dynamic stimulation leads to proteoglycan synthesis [43,44], resulting in an anabolic, protective response. In contrast, static, injurious, or high-magnitude mechanical stimuli inhibit aggrecan and type II collagen gene expression and induce catabolic activity [45,46]. The mechanotransduction process by which mechanical forces are transduced from the outside of the chondrocyte into biochemical signals leading to altered gene expression and protein production is slowly beginning to be understood. Integrins are recognised as being important in this process, but it is clear that molecules that modify integrin activation and downstream cell activity may have significant roles in regulation of chondrocyte responses to mechanical stimulation. In the present study, we have identified CD47/IAP as a further important accessory molecule for α5β1-integrin-dependent human articular chondrocyte mechanotransduction. CD47/IAP is expressed by chondrocytes in normal and osteoarthritic cartilage and is necessary for the integrin-dependent anabolic response of normal chondrocytes to mechanical stimulation. Chondrocytes from osteoarthritic cartilage, however, also demonstrate CD47/IAP-dependent mechanotransduction, although in these cells an anabolic response is not produced. As CD47/IAP interactions with integrins are important for the generation and specificity of downstream responses following integrin activation, it is possible that modulation of these interactions in disease may contribute to the abnormal phenotype and behaviour of osteoarthritic chondrocytes. It remains to be seen whether it will be possible to modulate chondrocyte mechanoreceptors such as CD47/IAP and integrins *in vivo*. If this is possible, it may be that mechanomimetics or small molecules can be developed that modify cellular mechanotransduction in such a way that catabolic mechanical stimuli would be inhibited or sensed as anabolic stimuli resulting in chondroprotective responses that will lead to slowing, or even reversal, of disease progression in OA.

## Abbreviations

BSA = bovine serum albumin; COMP = cartilage oligomeric matrix protein; GAPDH = glyceraldehyde-3-phosphate dehydrogenase; HRP = horseradish peroxidase; IAP = integrin-associated protein; IgV = immunoglobulin variable; OA = osteoarthritis; PCR = polymerase chain reaction; RGD = arginine-glycine-aspartic acid; SIRPα = signal-regulatory protein-alpha; TBST = Tris-buffered saline Tween-20; TSP = thrombospondin.

## Competing interests

The authors declare that they have no competing interests.

## Authors' contributions

MO and HSL (as part of their PhD studies) carried out the experiments on CD47/IAP expression by immunohistochemistry, co-immunoprecipitation, and Western blotting in addition to undertaking analysis of signalling events and changes in cell membrane potential following mechanical stimulation of chondrocytes. BG undertook some of the studies on the effects of mechanical stimulation and anti-CD47/IAP (as a visiting student) on chondrocytes and cell lines. SJMS co-supervised the work of MO, HSL, and BG and carried out the molecular analysis within the study. MOW undertook some electrophysiological experiments and supervised similar experiments by MO, HSL, and BG. FPL provided the CD47/IAP-null and transfected cell lines and was involved in the analysis of results of the studies on these cells. DMS conceived of the study, participated in its design and coordination, participated in the interpretation of results, and predominantly drafted the manuscript. All authors read and approved the final manuscript.
